# 모아의 환경적 건강에 대한 출산 코호트 효과: 체계적 고찰

**DOI:** 10.4069/kjwhn.2021.03.12

**Published:** 2021-03-20

**Authors:** JungMi Chae, Hyun Kyoung Kim

**Affiliations:** 1 Review & Assessment Research Department, HIRA Research Institute, Wonju, Korea; 1 건강보험심사평가원 심사평가연구실; 2 Department of Nursing, Kongju National University, Kongju, Korea; 2 국립공주대학교 간호학과

**Keywords:** Cohort studies, Environmental exposure, Environmental health, Pregnancy, Systematic review, 코호트 연구, 환경적 노출, 환경적 건강, 임신, 체계적 고찰

## Abstract

**Purpose:**

This study aimed to review recent findings from birth cohort studies on maternal and child environmental health.

**Methods:**

Birth cohort studies regarding environmental health outcomes for mothers and their children were investigated through a systematic review. A literature search was conducted in PubMed, CINAHL, the Cochrane Library, Embase, and RISS to identify published studies using the keywords using a combination of the following keywords: maternal exposure, environmental exposure, health, cohort, and birth cohort. Articles were searched and a quality appraisal using the Newcastle-Ottawa Scale for cohort studies was done.

**Results:**

A review of the 14 selected studies revealed that prenatal and early life exposure to environmental pollutants had negative impacts on physical, cognitive, and behavioral development among mothers and children up to 12 years later. Environmental pollutants included endocrine disruptors, air pollution (e.g., particulate matter), and heavy metals.

**Conclusion:**

This systematic review demonstrated that exposure to environmental pollutants negatively influences maternal and children’s environmental health outcomes from pregnancy to the early years of life. Therefore, maternal health care professionals should take steps to reduce mothers’ and children’s exposure to environmental pollutants.

## Introduction

### 연구 필요성

인류는 환경오염으로 기후 변화와 질병의 고통을 겪고 있으며, 환경오염 물질이 인체에 유해한 건강 결과로 나타나고 있다[1]. 환경적 건강(environmental health)은 인간 주변환경 조건과 영향력을 조절하여 건강을 유지, 증진하는 것을 의미하며, 환경오염에 따른 환경적 건강이 최근 주목을 받고 있다[2]. 환경적 건강이 취약한 인구 집단은 사회경제적 지위가 낮은 인구[3], 어린이[4], 여성, 특히 임신여성[5]이다. 사회경제적 지위가 낮은 인구는 환경오염이 더욱 심각한 지역에 거주하고 근무하여 건강에 유해한 영향을 받는다[3]. 어린이는 신체기관 성숙기라는 발달적 특성, 손을 입으로 가져가는 행위(hand to mouth), 기는(crawling) 행위의 특성, 작은 키로 오염 물질이 축적된 지표에 가까운 등의 이유로 건강에 유해한 영향을 받는다[4]. 여성은 에스트로겐 수용체가 많아 내분비계 장애물질(endocrine disruptors, EDCs)이 생식기관에 광범위한 영향을 미치고, 임신과 출산에 악영향을 미친다[5].

임부와 태아에 대한 환경적 위협의 결과는 속속 밝혀지고 있다. EDC가 임부에게 주는 영향을 살펴보면 임부의 비스페놀 A (bisphennol A, BPA), 프탈레이트(pthalate), 살충제 노출이 태아의 난포 수를 감소시켰다[6]. 식수가 파라벤(paraben)과 BPA 같은 EDCs로 오염된 경우 조산의 비율이 1.6배 높았다[7]. EDCs 노출은 태아의 정신운동 발달 문제와 생식 건강 문제를 유발하였으며[8], 프탈레이트는 태아의 대퇴골 길이와 신생아의 체중, 2–5세 아동의 체중을 감소시키는 영향을 미쳤다[9]. 임신 중에 살충제 성분이 노출되면 태아가 아동기가 되었을 때 인지적 발달에 부정적 영향을 미쳤다[10]. EDCs뿐 아니라 임신 초기에 전자기장에 노출된 임부의 경우 자연유산의 증가가 있었다[11]. 또한 중금속과 미세먼지도 임부와 태아의 건강에 영향을 주는 것으로 밝혀지고 있는데, 임부의 납 노출은 자연유산을 높였고[12], 미세먼지가 높은 지역의 임부는 조산과 자연유산 비율이 높았다[13].

환경적 건강은 어디에나 존재하는(ubiquitous) 환경오염 물질의 확인이 어려운 점, 장기간 노출과 중복 노출의 특성, 건강문제 발현의 장기적 특성, 동물실험이 아니기에 인간에게 추정되기 어려운 특징을 가지고 있다[6]. 따라서 환경오염 물질의 효과를 증명하기 위해서는 노출 인구집단의 종단적 코호트 연구가 적합하므로 세계적으로 환경적 건강에 관한 출산 코호트 연구가 이루어지고 있다[14]. 하지만 최근 세계적으로 10년간 활발히 이루어지는 출산 코호트 연구의 결과를 파악하기 위한 체계적 고찰 연구는 찾아보기 어렵다[14]. 그러므로 본 연구는 임신기간 환경오염 물질에 노출된 모아를 대상으로 건강에 미치는 영향을 장기간 연구해 온 출산 코호트 연구의 주제와 결과를 고찰하고, 환경오염 물질이 임부와 자녀의 건강에 어떠한 영향을 미쳤는지 유효성을 파악하고자 한다. 모아의 환경적 건강에 대한 출산 코호트 결과를 살펴봄으로써 인류에게 닥친 환경오염의 건강 영향을 직시하고, 환경적 건강상태를 증진하고 예방할 수 있는 분야를 파악하며, 취약한 모아를 보호하는 간호의 시각을 얻게 될 것이다.

### 연구 목적

본 연구의 목적은 모아의 환경적 건강에 대한 출산 코호트 연구를 체계적으로 고찰하고 유효성을 파악하는 것으로, 구체적인 목표는 다음과 같다.

첫째, 모아의 환경적 건강에 대한 출산 코호트의 주제를 파악한다.

둘째, 모아의 환경적 건강에 대한 출산 코호트의 결과를 파악한다.

셋째, 임부의 환경오염 물질 노출이 모아의 건강에 미치는 영향의 유효성을 파악한다.

## Methods

Ethical statements: This study received an ethical approval exemption from the Institutional Review Board of Kongju National University (KNU-IRB-2020-93).

### 연구 설계

본 연구는 모아의 환경적 건강에 대한 출산 코호트의 주제, 모아의 건강에 미치는 결과, 환경오염 물질 노출이 모아 건강에 미치는 영향의 유효성을 평가한 체계적 문헌고찰 연구이다. 고찰 과정은 National Evidence-based Collaborating Agency [15]의 체계적 고찰 지침에 따라 수행되었고 PRISMA (Preferred Reporting Items of Systemic Reviews and Meta-Analysis) [16] 보고 지침에 따라 작성하였다.

### 문헌 검색 전략

문헌 검색은 각 연구자가 모든 과정에서 case report를 작성하여 독립적으로 참여, 추출, 평가하였고 회의를 통하여 최종 선정하였다. 문헌 검색 전략을 확정하기 위한 검색은 2020년 4월 5일에서 2020년 5월 30일에 MeSH와 EMTREE의 통제어와 자연어의 유사어를 조합한 키워드로 수행하였다. 문헌 검색의 Participant Intervention Comparison Outcome Setting Time-Study Design (PICOST-SD)의 평가 질문으로 advance search를 수행하였다. 검색 키워드는 all text와 모든 기간 동안 영어로 된 peer review의 출판된 저널 논문으로 설정하였다. 검색원의 선정 기준은 미국국립의학도서관(National Library of Medicine)에서 제시한 COSI (Core Standard, Ideal)의 영역이었다[17]. 검색식은 PubMed에서 Mesh 검색을 이용하여 ((“Maternal Exposure”[Mesh] OR (“C”[TW] OR “Maternal Exposures” OR “Prenatal Exposures”[TW])) AND “Environmental Exposure”[Mesh] AND “Health”[Mesh] AND (“Cohort Studies”[Mesh] OR “Cohort”[TW] OR “Birth Cohort”[TW])와 Cochrane Library에서 [Maternal Exposure] explode all trees AND (Maternal exposure):ti,ab,kw, CINAHL에서 CINAHL Headings를 이용하여 (Maternal Exposure OR Prenatal Exposure OR Maternal Exposures OR Prenatal Exposures) AND (Environmental Exposure OR Environmental Exposures) AND Health AND (Cohort Studies OR Cohort OR Birth Cohort), Embase에서 EMTREE를 이용하여 (‘maternal exposure’/exp OR ‘prenatal exposure’/exp OR ‘environmental exposure’/exp) AND ‘health’/exp AND ‘cohort analysis’/exp를 적용하였다. 국내 문헌 검색은 RISS에서 ‘출생코호트 OR 출산코호트 AND 환경’의 키워드를 적용하였다. 연구의 비뚤림 감소를 위하여 biography search를 포함하였다.

문헌의 선정기준은 (1) 모아의 환경적 노출에 대한 건강 효과를 측정한 출산 코호트 연구, (2) 영어나 한국어로 된 연구, (3) 개별 출산 코호트 자료를 국가 간, 국가 내 데이터 뱅크로부터 합친 연구, (4) full text 접근이 가능한 연구, (5) peer review로 된 저널 문헌, (6) 출판된 연구, (7) 1968–2020년 사이의 연구였다. 문헌의 제외기준은 (1) 학위논문, (2) 학회 발표문, (3) 프로토콜 연구, (4) 서적, (5) 고찰 연구, (6) 보고서, (7) 연구 결과가 보고되지 않은 논문, (8) 회색 논문(conference abstract)이었다. 실험연구의 고찰에 사용하는 PICOST-SD의 핵심 질문 대신 환경적 건강문제에 관한 고찰에 사용되는 Participant Exposure Comparison Outcome Setting Time-Study Design (PECOST-SD)를 사용한 결과는 다음과 같다[18].

#### 대상(participants)

대상자의 선정기준은 건강 문제가 없는 임신 전체 기간의 임부로 하였으며 임부와 자녀를 한 쌍으로 한 연구를 포함하였다. 대상자의 제외기준은 임신 합병증을 가진 고위험 임부인 경우와 자녀만을 대상으로 한 연구는 제외하였다.

#### 노출(exposure)

환경오염 물질의 노출을 연구의 독립변인으로 한 연구를 선정하였고, 환경오염 물질 노출에는 EDCs, 미세먼지를 포함한 공기오염, 중금속, 전자파, 방사선을 선정하였다.

#### 비교 대상(comparison)

비교 대상은 코호트 연구의 설계에서 대조군을 설정한 연구와 환경오염 물질의 노출에 따른 종속변수의 차이 검정, 상관관계 분석, 회귀분석 등의 통계 결과가 제시된 연구를 포함하였다.

#### 결과(outcome)

연구 결과로 모체의 환경오염 물질 노출이 모체, 태아, 신생아, 영아, 유아, 학령전기, 학령기의 자녀에게 일으키는 모아 건강을 포함하였다. 건강문제로는 신체적, 인지적, 행동적, 정신적, 사회적 건강문제를 포함하였다. 청년기 이상의 자녀를 추적 조사한 연구는 발견되지 않았으므로 제외 기준이 되었다.

#### 장소(setting)

장소는 임부 관련 접근 가능한 자료로 지역사회, 의료기관, 웹사이트, 국가자료, 국제자료를 모두 포함하였다.

#### 평가시점(time)

평가시점은 임신 1, 2, 3기, 출생 시, 신생아기, 영아기, 유아기, 학령전기, 학령기에 해당하는 모아의 생애주기 중 일 회, 수 회의 추후평가를 모두 선정하였다.

#### 연구설계(study design)

연구설계는 코호트 연구만을 선정하였으며 전향적 코호트와 후향적 코호트를 모두 포함하였다. 코호트 연구를 위한 프로토콜 연구, 실험연구, 횡단적 조사연구, 질적 연구, 종설 및 문헌고찰 연구는 제외하였다.

### 분석문헌 선택과정

**Figure 1.**
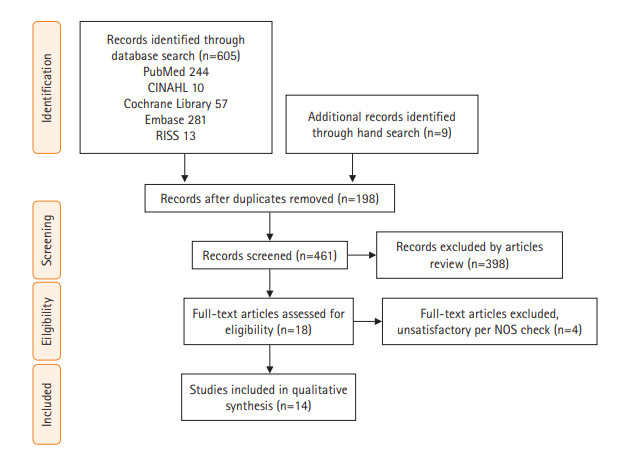
PRISMA flow diagram for the literature search. NOS: Newcastle-Ottawa Scale.

문헌 검색은 PubMed, CINAHL, Cochrane Library, Embase, RISS의 5개 검색 엔진으로부터 검색하였다. 검색 필터로 수집된 총 논문의 수는 605개(각각 244/10/57/281/13)였으며, hand search로 9편의 논문을 추가하였고, 중복 논문 198편이 제외되었다. 각 연구자는 독립적으로 총 416편의 논문의 초록을 읽어 이 중 선정기준에 적합하지 않은 398편을 제거하고 18편이 남았다. 18편 중 코호트 연구의 질 평가 도구로 추천되는[19] Newcastle-Ottawa Scale (NOS)를 사용하여[20] 각각의 질 평가 결과에 대한 연구 회의를 거쳐 4편을 제외한 최종 14편을 선정하였다[21-34] (Figure 1). 14편의 연구는 질적 고찰에 활용되었으며, 메타 분석에 활용할 수 있는 공통적 종속변수가 발견되지 않아 이질적 이었으므로 양적 유효성 평가는 수행하지 않았다.

### 문헌의 질 평가

논문의 질 평가 도구인 NOS는 호주의 Newcastle 대학과 캐나다의 Ottawa 대학이 협력하여 개발한 코호트 연구의 질 평가 도구로[20] 가장 흔히 사용되는 도구이다[19]. NOS는 코호트 선정(selection of cohorts), 코호트 비교(comparability of cohorts), 결과 사정(assessment of outcome)의 세 가지 평가 영역으로 구성되어 있다. 코호트 선정 영역은 노출 코호트의 대표성, 비노출 코호트의 선정, 노출의 확인, 연구시작 시 보여지지 않는 관심결과 제시의 4가지 세부 영역으로 이루어져 있으며, 세부 영역의 13개 답 가지 중 6개 답 가지의 질이 높은 항목에 별을 한 개씩 부여할 수 있고, 총 별의 수는 0–6의 범위를 가진다. 코호트 비교 영역은 연구설계나 분석의 기초한 코호트의 비교 1영역으로 이루어져 있으며, 2개의 항목에 응답하도록 되어 있고, 질이 높은 항목에 별을 한 개씩 부여할 수 있고, 총 별의 수는 0–2의 범위를 가진다. 결과 사정의 영역은 결과 사정, 결과 발생을 충분하게 오래 추후 관찰하였는지, 그리고 코호트 추후관찰의 적절성의 3가지 세부 영역으로 이루어져 있으며, 세부 영역의 10개 답 가지 중 5개 답 가지의 질이 높은 항목에 별을 한 개씩 부여할 수 있고, 총 별의 수는 0–5의 범위를 가진다. 별의 수가 많을수록 연구의 질이 높은 것을 의미하며, 3가지 영역에 대한 종합평가를 별의 수로 확인할 수 있다. 본 연구에서는 NOS 매뉴얼과 cohort star template를 사용하여[20] 각 연구자가 독립적으로 연구의 질 평가를 수행하고 결과를 대조하여 확정하였다. 불일치가 있는 항목은 연구자 간 회의를 거쳐 합의를 도출하였다(Table 1).

### 자료 분석

연구자들은 질 평가를 마친 선정된 최종 14편의 논문에 대하여 독립적으로 case report를 작성하여 정성적 분석을 시행하였다. 사례 보고의 항목은 출산 코호트 연구의 주제(저자, 연도, 국가, 코호트 고유명칭, 대상 인구집단, 모집 수/최종 분석 수, 모체 노출, 모아 결과, 추적 관찰 시점), 출산 코호트 연구의 결과(환경오염 물질, 결과변수, 연구 결과), 출산 코호트가 건강에 미치는 영향의 유효성의 질적 평가였다. Case report 작성 후 연구자 간 회의에서 일치도를 확인하였으며, 일치되지 않은 항목에 대하여 논문을 재평가하여 조정하였다.

## Results

### 연구의 질 평가 결과

14편 논문의 질 평가를 NOS 체크리스트로 확인한 결과[20] 코호트 선정 영역은 별 2–5의 범위를 가지고 있었고, 코호트 비교 영역은 별 1–2개, 결과 사정은 별 2–3개의 범위를 나타내어 질적인 평가 영역이 모든 연구에서 결손되지 않았다. 그러므로 14편의 연구가 모두 분석에 포함되었다. 코호트 선정의 4가지 세부 영역 중 노출의 확인이 모든 연구에서 확보되어 표본의 선정이 질적으로 충족되었다. 코호트 비교 영역은 연구설계나 분석에 기초한 코호트의 비교 영역이 모든 연구에서 최소한 1개 이상을 충족하여, 노출 정도를 비교하였거나 혼란변수의 조정이 있었음을 나타내었다. 결과 사정의 영역은 결과 사정, 결과 발생을 충분하게 오래 추후 관찰하였는지, 코호트 추후관찰의 적절성 중 적어도 2개 이상을 충족하여 결과 변수를 충분한 기간 사정하거나 결과의 근거를 명확하게 측정하였음을 나타내었다(Table 1).

### 모아의 환경적 건강에 대한 출산 코호트 주제

선정된 논문의 시기는 2014년이 2편[21,22], 2015년이 1편[23], 2016년이 2편[24,25], 2018년이 5편[26-30], 2019년이 3편[31-33], 2020년이 1편[34]으로, 2014년 이후 꾸준하게 연구되고 있었다. 국가별로는 유럽 연합이 3편[22,28,31]으로 가장 많았으며, 한국[21,34]과 프랑스[26,33]가 각각 2편, 중국[23], 푸에르토리코[24], 미국[25], 이탈리아[27], 노르웨이[29], 그리스[30], 벨기에[32]가 각각 1편이었다. 코호트는 고유 명칭을 모두 가지고 있었으며, 출산 코호트의 대상자 특징을 사용한 약어[21,22,24,26-34], 환경오염 물질 명칭[25], 지역 명칭[23]을 사용하였다. 대상 인구집단은 자녀의 성별과 관련 없이 모아의 짝을 지은 연구가 12편[21-23,25-32,34], 모자(mother-son)를 인구집단으로 한 연구가 1편[33], 임신기간 모체를 인구집단으로 한 연구가 1편[24]이었다. 인구집단의 모집 수는 179명[24]에서 31,472명[28]이었고, 최종 분석 수는 106명[24]에서 10,542명[23]이었다. 모체 노출 환경오염 물질은 EDCs가 6편[22,24,25,28,29,33], 공기오염이 5편[26,27,31,32,34], 중금속이 3편[21,28,34]가 있었으며, 기타 요리 연료가 1편[23], 흡연과 수유가 1편이었다[30]. 전자파와 방사선 노출 효과를 다룬 연구는 없었다. 이 중 중금속과 EDCs를 함께 측정한 연구[28]와 중금속과 공기오염을 함께 측정한 연구가 있었다[34]. 모아 결과 변수로는 자녀의 신체적 발달이 3편[21,33,34], 출생 시 체중이 2편[22,23], 자녀의 인지, 신경, 행동발달이 2편[30,34], 자녀의 호르몬 수치가 1편[24], 자녀의 폐 기능이 1편[26], 자녀의 비만이 1편[27], 자녀의 EDCs와 중금속 수치가 1편[28], 자녀의 면역 억제가 1편[29], 자녀의 세포노화도가 1편[31], 자녀의 혈압이 1편[32]이었다. 첫 추적 관찰 시점은 임신 16주가 1편[24], 출생 시가 5편[22,23,32-34], 출생 후 6개월이 2편[21,25], 출생 후 1년이 1편[31], 출생 후 2년이 1편[29], 4년이 2편[27,30], 6년이 1편[28], 8년이 1편[24]이었고, 주로 출생 시부터 출생 후 1년 이내의 시기가 대부분(8편) [21-23,25,31-34]이었다. 마지막 추적 관찰 시점은 출생 시가 4편[22,23,32,33]이었고, 임신 28주[24], 출생 후 6개월[25], 출생 후 1년[31], 2년[21], 4년[30], 6년[34], 8년[27], 9년[26], 10년[29], 12년[28]이 각각 1편이었다. 추적 관찰 회수는 1–6회로, 대부분 1회(8편) [22,23,25,26,30-33]이었고, 2회가 4편[24,27-29], 3회가 2편[21,23], 8회가 1편[34]이었다(Table 2).

### 모아의 환경적 건강에 대한 출산 코호트 결과

노출 환경오염 물질은 구체적으로 다이옥신, 프탈레이트, 페놀, BPA 등 40개 이상의 EDCs 6편[22,24,25,28,29,33], 미세먼지 등의 공기오염 5편[26,27,31,32,34], 납, 수은, 망간, 카드뮴 등의 중금속 2편[21,34], 연료(석탄, 나무땔감, 전기스토브) 1편[23], 질소화합물, 흡연 1편[30]이 있었다. 모체의 결과변수는 구체적으로 제태기간 1편[22], 태반 무게 1편[33], 에스트로겐, 프로게스테론, 성호르몬 결합 글로불린, 갑상선 자극호르몬, 갑상선 호르몬 1편[24]이었다. 자녀의 결과변수는 출생 시 체중과 신장 5편[21-23,27,33], 머리둘레, 허리둘레 2편[27,34], 태반과 출생 시 체중의 비율 1편[33], 결핵반응 면역글로불린 1편[25], 폐기능 1편[26], 비만도 1편[27], 혈중 고밀도지질 1편[27], 백혈구의 텔로미어 길이(leukocyte telomere length, LTL) 1편[31], 혈압 1편[32], 40개의 화학물질 체내 농도 1편[28], 아토피 피부염, 비염, 천식, 폐쇄성 호흡기 질환, 알러지, 호흡기 감염 1편[29], 주의력 결핍 과잉행동 장애 1편[30], 행동문제, 신체발달지수, 지능, 자폐행동 1편[34]이 있었다(Table 3).

### 임부의 환경오염 물질 노출이 모아의 건강에 미치는 영향의 유효성

연구 결과 EDCs의 노출 효과를 살펴보면, 모체의 다이옥신 노출이 짧은 제태기간[22], 출생 시 자녀의 저체중[22]과 관련이 있었고, 프탈레이트와 페놀 노출이 태반 무게 감소와 관련이 있었다[33]. 임부의 프탈레이트 노출은 태반과 출생 시 신생아 체중 사이의 비율 감소와 관련이 있었다[33]. 페놀과 파라벤 노출은 모체의 성호르몬 결합 글로불린을 증가시키고[24], 에스트로겐과 프로게스테론을 감소시켰으며[24], 에스트라디올과 프로게스테론의 비율을 감소시켰다[24]. 자녀에게는 임부의 바이페닐과 디클로로 에틸렌 노출이 자녀의 결핵 백신 글로불린의 효과를 낮추어 항체 형성을 감소시켰다[25]. 40개의 모체 EDCs 농도는 자녀의 EDCs 농도와 관련이 있었고[28], 90% 이상의 물질이 모체에게서 더 높은 농도로 나타났다[28]. 임부의 과불화화합물(perfluorinated alkylated substances) 노출은 자녀의 면역 억제, 상기도염, 하기도염과 관련이 있었다[29].

미세먼지를 포함한 공기오염 물질의 노출 효과를 살펴보면, 임부의 미세먼지 노출[27,34], 임신 시 석탄이나 나무 땔감을 연료로 사용하는 것이 출생 시 저체중[23]과 관련이 있었다. 이산화질소 노출은 자녀의 폐기능 중 강제폐활량(forced vital capacity)을 낮추었고[26] 반복된 하기도 감염과 알러지의 민감성을 높였다[26]. 임부의 미세먼지, 질소화합물 노출은 자녀의 비만과는 통계적으로 유의한 관계가 없었다[27]. 임부의 흡연은 자녀의 낮은 인지점수와 관련이 있었다[30]. 임부의 미세먼지 노출은 자녀의 LTL 감소와 관련이 있었다[31]. 임부의 이산화질소 노출은 신생아의 높은 수축기, 이완기 혈압과 관련이 있었다[32].

중금속 노출 효과를 살펴보면, 임부의 납, 수은, 카드뮴, 망간 노출은 자녀의 낮은 체중 증가[21,34], 낮은 인지발달 점수, 정신발달 점수, 신체발달 점수, 신경발달 점수와 관련이 있었고[34], 높은 자폐행동과 행동문제 점수와 관련이 있었다[34] (Table 3).

## Discussion

본 연구는 체계적 고찰 방법에 의해 14편의 환경오염 물질 노출이 모아의 건강문제에 영향을 미치는지 조사한 출산 코호트 연구들의 주제와 결과를 파악하고 모아 건강에 미치는 영향을 분석하였다. 14편의 결과 변수는 모아의 신체적, 인지적, 행동적 건강 상태에 통계적으로 유효한 영향을 주는 것으로 나타났다.

본 연구의 강점은 세계적으로 환경오염 물질이 모아의 건강에 미치는 영향에 관심을 가지고 출산 코호트가 국가단위 사업으로 활발히 수행되고 있는 시점에서, 연구 결과들을 통합하여 살펴 볼 수 있도록 체계적인 고찰이 이루어졌다는 점이다. 출산 코호트는 환경적 건강을 포함하여 다양한 모아 건강을 주제로 최근 10년 사이 유럽을 중심으로 111개 이상 활발히 진행되고 있는데[14], 1−10년 가량의 연구 기간을 두고 진행되는 출산 코호트 연구의 특성상 결과 변수를 확인하기에 적절한 시점에 도달하였다. 본 연구 결과 모아의 환경적 건강 관련 코호트는 2014년 이후 유럽 이외에도 한국과 중국을 포함하여 다수 국가에서 이루어지고 있었으며, 연구의 규모도 매우 커서 대상자의 수가 1,000명 이상인 경우가 대다수였다. 본 고찰 논문들은 NOS 질 평가 결과인 코호트 선정, 코호트 비교, 결과 사정 영역이 질적으로 우수한 것으로 나타나[20], 모아 건강의 시사점을 제공하는 근거기반 확립에 기여하였다. 그러므로 출산 코호트 자료 결과를 통합하여 제시하면 임신 중 환경오염 물질 노출로 인한 모아의 환경적 건강 결과 관련 지식을 확장하는 데 도움을 줄 수 있다[14].

본 연구 결과 모아의 환경적 건강에 대한 출산 코호트 주제는 EDCs, 공기오염, 중금속이 임신 기간 동안 모체에 축적되어 모체와 출산한 자녀에게 어떠한 건강 영향을 주었는지 조사한 전향적 코호트 연구라고 요약할 수 있다. 환경오염 물질별로 결과를 고찰하면 다음과 같이 논의할 수 있다.

첫째, 연구물 6편으로 가장 많은 환경오염 노출물질로 조사된 EDCs는 호르몬의 생산, 방출, 대사, 전달, 연결, 활동, 제거를 방해하는 물질로 정의되고 있으며, 적은 양으로도 효과를 나타내어 환경호르몬이라 부른다[35]. 임신기간은 EDCs에 취약한 시기로 태아에게 모체의 생활습관이 전달되고, 모유 수유를 통하여 물질을 전달하게 되어 미래 세대에 영향을 미치는 소리 없는 유해물질이다[35]. 본 연구에서는 다이옥신, BPA, 프탈레이트, 트리클로산, 페놀, 벤조페논, 파라벤, 과불화화합물 등을 임부의 소변, 혈액, 임부가 마시는 식수, 제대혈, 자녀의 소변에서 농도를 검사하여[22,24,25,28,29,33] 생리적 지표로 활용하였으므로, 임부의 EDCs 노출을 객관적으로 평가할 수 있었다는 장점이 있었다.

둘째, 연구물 5편으로 두 번째로 많은 공기오염 물질은 직경이 10 µm 이하인 미세먼지 particulate matter 10 (PM_10_)과 직경이 2.5 µm이하인 초미세먼지 PM_2.5_로 주로 측정하며, 배기가스로 인한 주 오염원인 이산화질소 NO_2_ (nitrogen dioxide), 질소화합물 NOx (nitrogen dioxides), 탄소(black carbon)로 측정하였다[26,27,31,32,34]. Jiang 등[23]의 연구에서는 임신기간 사용한 요리 연료가 땔감, 석탄, 전기 스토브인지 공기오염을 설문 조사하였고, 5편의 연구물들에서는[26,27,31,32,34] 오염 물질의 농도를 측정하기 위하여 인공위성 대기 질 정보, 도로교통 정보의 오염 물질 농도, 거주지에서부터 주요 도로와의 거리 등의 측정법을 사용하였다. 이들 연구물에서는 거주지의 대기 질 측정을 하였으므로, 간접적이기는 하나 객관적 지표로 평가하였다는 장점이 있다.

셋째, 연구물 3편에서 환경오염 물질로 측정한 임부의 중금속 노출은 납이 가장 많았고, 수은, 망간, 카드뮴에 관심을 가지고 연구하였다[21,28,34]. 유럽의 출산 코호트는 국가 간 자료를 바이오뱅크에서 통합 관리하고 있어, 5, 6개 국가가 연합하여 관심 노출물질과 결과지표를 활용하며 공유하고 있다. HELIX (Human Early Life Exposome)가 유럽연합 6개 국가 코호트들의 중금속 노출과 모아의 생리적 지표를 활용하였으며[28], 한국의 출산 코호트인 MOCEH (Mothers and Children’s Environmental Health Study)에서도[21,34] 전국 개별 코호트들이 임부의 혈중 납, 수은 등의 중금속 농도와 제대혈 중금속 농도 측정결과 등을 활용하였다.

본 연구에서 고찰된 모아의 환경적 건강에 대한 출산 코호트의 결과는 모체와 자녀의 부정적 건강으로 도출되었다. 모아의 건강 결과에 초점을 맞추어 논의하면 다음과 같이 요약할 수 있다. 첫째, 임부의 환경오염 물질 노출은 모체의 제태기간 감소, 태반 무게 감소, 성호르몬 감소, 성호르몬 결합 글로불린 감소의 신체적 건강문제와 관련이 있다는 점이다. 둘째, 자녀에게는 임부의 환경오염 물질 노출이 저체중, 폐기능 감소, 상기도염 증가, 하기도염 증가, 백신 항체 형성 감소, 면역력 감소, 민감성 증가, 혈압의 증가, LTL 감소와 같은 부정적 신체 결과와 관련이 있다는 것이다. 셋째, 임부의 환경오염 물질 노출은 자녀에게 인지 기능, 정신발달 기능, 신경발달 기능, 행동발달 기능의 부정적 발달과 관련이 있다는 점이다.

첫째, 모체의 신체적 결과를 살펴보면 덴마크, 그리스, 노르웨이, 스페인, 영국의 NewGeneris (Newborns and Genotoxic exposure risks) 코호트에서[22] 다이옥신 노출이 제태기간을 0.4주 감소시켰고, 자녀가 남아인 경우에 더 큰 영향을 주었으므로 임부의 다이옥신 노출은 조산의 위험요인으로 작용할 수 있음을 시사한다. 프랑스의 EDEN (Etude des Déterminants pré et postnatals du développement et de la santé de l’Enfant) 코호트에서는 임신 23–29주에 소변 내 트리클로산과 프탈레이트의 농도를 측정하고 출산 시 태반의 무게에 미치는 영향을 조사한 결과, EDCs 농도 1단위 증가 시 태반 무게가 각각 4.11 g과 10.9 g이 감소하였으므로, 임부의 화학물질 노출이 태반 무게를 감소시킬 수 있음을 알 수 있다[33]. 또한 푸에르토리코의 PROTECT (Puerto Rico Test for Exploring Contamination Threats)에서는 임부의 부틸파라벤 증가로 에스트라디올이 8.46% 감소하고, 메틸파라벤 증가로 성호르몬 결합 글로불린이 7.7% 증가하였으므로, 임부의 화학물질 노출이 모체의 호르몬 변화를 가져올 수 있음을 알 수 있었다[24].

둘째, 자녀의 신체적 결과는 더 많은 연구가 이루어졌고 특히 출생 시 신생아 저체중을 보이는 결과가 많았으므로[21-23,33,34], 임신 중의 환경오염 물질 노출은 출생 시부터 성장과정에 부정적인 결과를 가져온다고 할 수 있다. 국내 연구에서도 BPA 노출이 태아 대퇴골 길이(femur length)를 0.06 cm 감소시키고, 납 노출이 신생아 체중을 감소시키는 것으로 나타났다[34,36]. 중국 연구에서는 전기 연료에 비해 나무 땔감을 연료로 사용하는 임부의 자녀에게서 저체중아 비율이 2.51배 높게 나타나[23], 빈곤과 가사노동으로 인한 실내 공기오염에 여성이 취약하며, 자녀 세대에까지 영향을 주어 환경의 신체적 짐(body burden)이 임부에게 부과됨을 알 수 있다[37]. 임부의 과불화화합물 농도가 2–10세 자녀의 아토피 피부염과 폐질환 증가와 관련이 있으며, 제대혈에서 과불화화합물 농도가 높고 상기도염과 하기도염 발생이 높은 것으로 나타나 EDCs의 노출이 자녀의 호흡기 질환과 면역력 감소에 영향이 있음을 알 수 있었다[29]. 40개 이상의 EDCs 농도를 임부와 자녀 사이에서 비교한 결과 90% 이상에서 어머니보다 자녀에게 높은 농도로 나타났고, 그 수치가 독일 환경부 농도 기준을 초과한 것을 보아 모아의 환경오염 물질 노출 정도는 심각함을 알 수 있다[28]. 자녀의 공기오염 물질 노출과 상기도, 하기도염, 폐기능 저하의 관련성 대한 근거도 제시되었다[26,29]. 임신 2기의 질산가스 노출은 7–8세 된 자녀의 반복된 하기도염과 알러지 민감성을 높이며 강제날숨유량(forced expiratory flow)를 25–75% 감소시키는 것으로 나타나 호흡기계 감염 및 폐기능 저하가 장기적으로 관련됨을 알 수 있다[29].

자녀에게 미치는 건강 결과는 코호트 연구 기간이 최대 12년까지 이루어진 것을 보아 임부의 환경오염 물질 노출의 결과가 자녀 세대의 건강에 미치는 영향이 장기적이다[28]. 더욱이 임부의 질산 노출이 8세 자녀의 혈액의 DNA 검사 결과 백혈구의 세포 노화 마커인 LTL을 1.5% 감소시켰다는 세포학적 결과를 보아[31] 부정적 파급이 영구적 영향을 미칠 수도 있음을 알 수 있다. 환경오염 물질로부터의 자녀 건강에 보호작용을 간접적으로 확인할 수 있는 바로는 큰 도로로부터의 거리가 2배 멀어질수록 LTL의 길이가 1.6% 증가하였다는 점이 있다[31]. 또 다른 연구에서는 거주지 5 km 이내에 녹지가 있는 경우 자녀의 수축기 혈압이 1.2 mmHg 감소하고, 이완기 혈압도 1.2 mmHg 감소하여 보호 효과가 있는 것으로 나타났다[32]. 임부의 납 노출은 24개월 아기의 체중을 0.28 kg 감소시키고 신장을 0.51 cm 감소시켜 신체 성장에 영향을 미치는 것으로 나타났으나, 임부가 칼슘 섭취를 한 경우 보호 효과가 나타났으므로 임부의 식이에 따라 자녀의 건강 보호 효과가 나타날 수 있음을 알 수 있다[21]. 임부가 흡연을 하지 않고, 모유 수유를 한 경우에도 자녀의 주의력결핍과잉행동 증후군 점수가 낮게 나타나 임부의 건강이 자녀의 건강 결과에 보호작용을 하게 되므로[30], 임부의 생활습관과 건강행위가 자녀의 환경적 건강상태를 향상시킬 수 있을 것이다.

셋째, 자녀의 인지적 성장과 발달에 미치는 영향이 추적기간에 종단적으로 이루어져 임부의 환경오염 물질 노출이 자녀의 신경정신 발달에 부정적임을 확인할 수 있었다. 임부의 납, 수은, 망간, 카드뮴, 미세먼지, 프탈레이트 노출은 출생 후 6세까지 정신발달 점수, 신경발달 점수, 행동문제 점수, 지능 점수, 자폐행동 점수에 악영향을 미치고 있었다[34]. 임신 중 살충제 성분인 유기염소에 노출이 되면 신생아의 머리 둘레가 적고, 인지기능이 저하되면 신경발달이 저해된다는 연구 결과도 같은 맥락이다[10]. 모아보건 전문가는 환경오염이 자녀의 호흡기, 면역체계, 신체 성장은 물론이고 신경발달의 문제도 초래할 수 있다는 연구 결과에[30,34] 주목할 필요가 있다. 독일의 어린이 코호트인 KUNO-Kids에서는 어린이의 환경오염 물질 노출뿐 아니라 가족의 생활환경을 다각도로 분석하기 위해 5,000–10,000 가족의 사회인구학적 특성, 임신 시 생활습관, 건강행위, 의학적 기록, 가정환경, 정신적 상태를 광범위하게 장기 추적하고 있으며, 세포의 손상을 일으키는지 분석하고 있다[37]. 그러므로 임부의 환경오염 물질 노출이 자녀의 발달 문제를 일으키는지 장기적 전향적 코호트 연구로서 세포학적 근거가 보강될 것으로 보인다[37].

본 연구의 의의는 임부의 환경오염 물질에 대한 건강결과에 대하여 통합하여 제시함으로써 환경적 건강 및 모아보건 전문가에게 임부 출산교실 등의 중재에 보건 실무에 적용할 수 있는 근거를 다양하게 활용할 수 있는 자료를 제공하였다는 점이다. 모아 간호 연구자에게는 임부의 환경적 건강에 대한 지식과 인식을 민감하게 가질 수 있고, 미래 연구 방향에 영감을 부여할 수 있으며 연구 가이드라인의 역할을 할 수 있다는 점이다. 모아 간호 교육자들은 최근 부상하고 있는 환경적 건강 영역에 대한 통합적 제시로 전통적 모아 간호의 영역에 환경적 건강 영역이 필요함을 인식할 수 있으며, 환경정책 전문가에게는 임부의 환경적 건강이 미래 세대에 미치는 영향을 고려하여 환경오염 물질로부터 임부를 보호할 수 있는 정책적 함의를 제공하였다는 점이다. 그러므로 모아보건 전문가는 임부의 환경오염 물질 노출인 EDCs, 공기오염 물질, 중금속 등의 부정적 모아 건강결과를 인식하고 개선해 나갈 필요가 있다.

본 연구의 제한점은 체계적 고찰 연구로 영어와 한국어로 된 문헌만을 대상으로 하여 선정 오류가 있을 수 있으며, 출산 코호트가 전 세계적으로 널리 연구되어 있어 출판되지 않고 진행 중인 연구들에 대한 분석이 결여되었다는 점이다. 또한 유럽의 출산 코호트 연구는 노출인자와 결과변수에 따라 다양한 조합으로 코호트 고유명칭을 변경하며 사용하고 있어, 통합된 결과에서 중복 데이터가 포함되어 있을 가능성이 있다는 점이다. 결과변수의 다양성으로 인하여 메타분석이 불가능하였다는 제한점도 있다.

미래의 코호트 연구에는 임부의 EDC를 피하는 건강행위와 식습관 등의 생활양식을 반영하여 모체와 자녀의 건강에 미치는 영향을 조사하는 것을 제안한다. 또한 본 연구에서와 같이 다양한 생·물리학적 지표를 활용하여 소변 내, 혈중, 제대혈, 모유의 EDCs 농도, 대기 질 지표를 활용한 미세먼지와 질산 농도, 큰 도로와의 거리, 거주지의 녹지 비율 등을 측정하고, 자녀의 건강 지표로는 출생 시 체중, 신장 등의 객관적 지표를 활용하여 보다 높은 수준의 근거를 확보하는 것을 제안한다. 또한 전향적 연구를 통하여 추적조사가 장기적으로 이루어져 미래 세대에 미치는 영향을 조사할 수 있도록 연구를 설계하고 프로토콜 연구 단계를 거쳐 기획할 것을 제언한다.

## Figures and Tables

**Table 1.** Quality appraisal using the Newcastle-Ottawa Quality Assessment Form for selected cohort studies (N=14)

Reference	First author	Publication year	Selection	Comparability	Outcome	Quality^†^
[21]	Hong	2014	****	**	***	Good
[22]	Vafeiadi	2014	*****	**	***	Good
[23]	Jiang	2015	**	**	****	Fair
[24]	Aker	2016	***	**	***	Good
[25]	Jusko	2016	***	**	***	Good
[26]	Bougas	2018	****	**	***	Good
[27]	Fioravanti	2018	***	**	***	Good
[28]	Haug	2018	***	*	**	Good
[29]	Impinen	2018	*****	**	***	Good
[30]	Kampouri	2018	***	**	***	Good
[31]	Clemente	2019	***	*	**	Good
[32]	Madhloum	2019	**	**	***	Fair
[33]	Philippat	2019	**	*	***	Fair
[34]	Shah	2020	****	*	***	Good

† Good quality: 3 or 4 stars in the selection domain AND 1 or 2 stars in the comparability domain AND 2 or 3 stars in the outcome/exposure domain; fair quality: 2 stars in the selection domain AND 1 or 2 stars in the comparability domain AND 2 or 3 stars in the outcome/exposure domain; poor quality: 0 or 1 star in the selection domain OR 0 stars in the comparability domain OR 0 or 1 stars in the outcome/exposure domain.

**Table 2.** Characteristics of birth cohort studies (N=14)

Reference	First author	Selected	Country	Title of cohort	Study design	Participant	Recruit/analysis (n)	Maternal exposure	Maternal or infantile outcome	Follow-up
[21]	Hong	2014	Republic of Korea	MOCEH	Prospective cohort	Mother-child pairs	1,475/1,150	Heavy metal	Physical development	At 6, 12, and 24 months
[22]	Vafeiadi	2014	Europe (5 countries)^†^	NewGeneris	Prospective cohort	Mother-child pairs	967/269	Chemicals	Gestational age and weight	At birth
[23]	Jiang	2015	China	Lanzhou cohort	Prospective cohort	Mother-child pairs	14,359/10,542	Cooking fuel	Birth weight	At birth
[24]	Aker	2016	Puerto Rico	PROTECT	Prospective cohort	Pregnant women	179/106	Chemicals	Hormonal level	At 16–20 and 24–28 weeks of gestation
[25]	Jusko	2016	United States	PCB	Prospective cohort	Mother-child pairs	1,134/973	Chemicals	Antibody level	At 6 month
[26]	Bougas	2018	France	PARIS	Prospective cohort	Mother-child pairs	3,840/788	Air pollution	Lung function	At 8–9 years
[27]	Fioravanti	2018	Italy	GASPII	Prospective cohort	Mother-child pairs	719/581	Air pollution	Obesity	At 4 and 8 years
[28]	Haug	2018	Europe (6 countries)^‡^	HELIX	Prospective cohort	Mother-child pairs	31,472/1,301	Chemicals and heavy metals	Chemicals and heavy metals	Between 6 and 12 years
[29]	Impinen	2018	Norway	ECA	Prospective cohort	Mother-child pairs	3,754/641	Chemicals	Immunosuppression	At 2 and 10 years
[30]	Kampouri	2018	Greece	RHEA	Prospective cohort	Mother-child pairs	1,363/849	Smoking and feeding	Cognitive behavior	At 4 year
[31]	Clemente	2019	Europe (6 countries)^‡^	HELIX	Prospective cohort	Mother-child pairs	31,472/1,396	Air pollution	Cell aging	At 1 year
[32]	Madhloum	2019	Belgium	ENVIRONAGE	Prospective cohort	Mother-child pairs	762/427	Air pollution	Blood pressure	At birth
[33]	Philippat	2019	France	EDEN	Prospective cohort	Mother-son pairs	998/473	Chemicals	Physical development	At birth
[34]	Shah	2020	Republic of Korea	MOCEH	Prospective cohort	Mother-child pairs	1,751/442	Heavy metals and air pollution	Physical, neurologic, and behavioral development	At birth, 6 month, 1, 2, 3, 4, 5, and 6 years

ECA: Environment and Children Asthma; EDEN: Etude des Déterminants pré et postnatals du développement et de la santé de l’Enfant; ENVIRONAGE: ENVIRonmental influence ON early AGEing; GASPII: Gene and Environment Prospective Study in Infancy in Italy; NewGeneris: Newborns and Genotoxic exposure risks; HELIX: Human Early Life Exposome; MOCEH: Mothers and Children’s Environmental Health Study; PARIS: Pollution and Asthma Risk; PCB: polychlorinated biphenyls; PROTECT: Puerto Rico Test for Exploring Contamination Threats.

† Denmark, Greece, Norway, Spain, and England;

‡ England, France, Spain, Lithuania, Norway, and Greece.

**Table 3.** Outcomes of birth cohort studies (N=14)

Reference	First author	Selected	Pollutant	Outcome measure	Conclusion
[21]	Hong	2014	Lead	Infants’ weight and height	Lead exposure was associated with low infantile weight and height.
[22]	Vafeiadi	2014	Dioxin-like plasma activity	Birth weight and gestational age	Dioxin exposure was associated with low birth weight and short gestational age.
[23]	Jiang	2015	Coal, biomass, and electromagnetic stove	Birth weight	Using coal and biomass as cooking fuel were associated with low birth weight.
[24]	Aker	2016	BPA, BP-3, 2,4-DCP, 2,5-DCP, TCS, MPB, BPB, and PPB	Estradiol, progesterone, estradiol/progesterone, SHBG, TSH, FT3, and FT4	Phenol exposure was associated with decrease of progesterone. Paraben exposure was associated with increase of SHBG and decrease of estrogen and progesterone.
[25]	Jusko	2016	PCB-153 and DDE	BCG-immunoglobulin G and A	PCB and DDE exposure was associated with low BCG-immunoglobulin G and A.
[26]	Bougas	2018	NO_2_	Pulmonary function test index	NO_2_ exposure was associated with low forced vital capacity (FVC).
[27]	Fioravanti	2018	NO_2,_ NOx, PM_10_, and PM_2.5_	Weight, height, waist, hip circumference, body mass index, and high-density lipoprotein	NO_2,_ NOx, PM_10_, and PM_2.5_ exposure was not associated with weight, height, waist, hip circumference, BMI, and HDL.
[28]	Haug	2018	40 chemicals and heavy metals	40 chemicals and heavy metals	For persistent compounds and heavy metals correlations between maternal exposure and child outcomes were moderately high.
[29]	Impinen	2018	PFAS (PFUnDA, PTOS, PFOA, PFOSA, and PFNA)	Atopic dermatitis, pulmonary function, rhinitis, asthma, obstructive airway disease, allergy, and respiratory infection	PFAS exposure was associated with high atopic dermatitis, rhinitis, asthma, allergy, respiratory tract infections, and reduce pulmonary function.
[30]	Kampouri	2018	Television watching, smoking, and breast feeding	Attention deficit hyperactivity disorder test	No smoking and breastfeeding were associated with low attention deficit hyperactivity disorder score.
[31]	Clemente	2019	NO_2_ and PM_2.5_	Leukocyte telomere length	NO_2_ and PM_2.5_exposure was associated with short telomere length.
[32]	Madhloum	2019	NO_2,_ black carbon, PM_10_, and PM_2.5_	Systolic and diastolic blood pressure	Air pollution was associated with high systolic and diastolic blood pressure.
[33]	Philippat	2019	Phthalate and phenol in urine	Ratio of placenta and birth weight	Phthalate exposure was associated with low placental weight and placental-to-birth weight ratio.
[34]	Shah	2020	Lead, mercury, manganese, cadmium, and PM	Head circumference, weight, mental and physical development index, behavioral problems, intelligent quotation, autistic behavior, and atopic dermatitis	Heavy metal and air pollutant exposure was associated with low birth weight, low cognition, high atopic dermatitis, and high behavioral problems.

BPA; bisphenol A; BPB; bisphenol B; BPF: bisphenol-F; BP-3; benzophenone-3; 2,4-DCP: 2,4-dichlorophenol; 2,5-DCP: 2,5-dichlorophenol; DDE: 1,1-dichloro-2,2-bis (p-chlorophenyl) ethylene; FT3: free triiodothyronine; FT4: free thyroxine; MPB: methylparaben; NO_2_: nitrogen dioxide; NOx: nitrogen oxides; PCB: polychlorinated biphenyls; PFAS: perfluoralkyl substance; PFOA: perfluorooctanoic acid; PFNA: perfluorononanoic acid; PFOS: perfluorooctane sulfonic acid; PFOSA: perfluorooctanesulfonamide, PFUnDA: perfluoroundecanoic acid; PM: particulate matter; PPB: propylparaben; SHBG: sex-hormone-binding globulin; TCS: triclosan; T4: total thyroxine; TSH: thyroid-stimulating hormone.
